# Detection of Ancestry Informative HLA Alleles Confirms the Admixed Origins of Japanese Population

**DOI:** 10.1371/journal.pone.0060793

**Published:** 2013-04-05

**Authors:** Hirofumi Nakaoka, Shigeki Mitsunaga, Kazuyoshi Hosomichi, Liou Shyh-Yuh, Taiji Sawamoto, Tsutomu Fujiwara, Naohisa Tsutsui, Koji Suematsu, Akira Shinagawa, Hidetoshi Inoko, Ituro Inoue

**Affiliations:** 1 Division of Human Genetics, Department of Integrated Genetics, National Institute of Genetics, Mishima, Shizuoka, Japan; 2 Division of Molecular Life Science, School of Medicine, Tokai University, Isehara, Kanagawa, Japan; 3 GenoDive Pharma Inc. Isehara, Kanagawa, Japan; 4 Clinical Pharmacology, Clinical Data Science Department, Takeda Development Center Japan Takeda Pharmaceutical Co, Ltd. Chuo-ku Osaka, Japan; 5 Clinical Pharmacology, Astellas Pharma Global Development Astellas Pharma Inc. Itabashi-ku, Tokyo, Japan; 6 PGx office, Department of Clinical Research and Development, Otsuka Pharmaceutical Co., Ltd. Chuo-ku, Osaka, Japan; 7 Clinical Pharmacology Department, Development Division Mitsubishi Tanabe Pharma Corporation Yodogawa-ku, Osaka, Japan; 8 PGx, Clinical Research Taisho Pharmaceutical Co., Ltd. Toshima-ku, Tokyo, Japan; 9 Translational Medicine and Clinical Pharmacology Department, R&D Division Daiichi-Sankyo Co., Ltd. Shinagawa-ku, Tokyo, Japan; University of Cagliari, Italy

## Abstract

The polymorphisms in the human leukocyte antigen (HLA) region are powerful tool for studying human evolutionary processes. We investigated genetic structure of Japanese by using five-locus HLA genotypes (*HLA-A*, *-B*, *-C*, *-DRB1*, and *-DPB1*) of 2,005 individuals from 10 regions of Japan. We found a significant level of population substructure in Japanese; particularly the differentiation between Okinawa Island and mainland Japanese. By using a plot of the principal component scores, we identified ancestry informative alleles associated with the underlying population substructure. We examined extent of linkage disequilibrium (LD) between pairs of HLA alleles on the haplotypes that were differentiated among regions. The LDs were strong and weak for pairs of HLA alleles characterized by low and high frequencies in Okinawa Island, respectively. The five-locus haplotypes whose alleles exhibit strong LD were unique to Japanese and South Korean, suggesting that these haplotypes had been recently derived from the Korean Peninsula. The alleles characterized by high frequency in Japanese compared to South Korean formed segmented three-locus haplotype that was commonly found in Aleuts, Eskimos, and North- and Meso-Americans but not observed in Korean and Chinese. The serologically equivalent haplotype was found in Orchid Island in Taiwan, Mongol, Siberia, and Arctic regions. It suggests that early Japanese who existed prior to the migration wave from the Korean Peninsula shared ancestry with northern Asian who moved to the New World via the Bering Strait land bridge. These results may support the admixture model for peopling of Japanese Archipelago.

## Introduction

The human leukocyte antigen (HLA) region is the human equivalent of the major histocompatibility complex (MHC), which spans approximately 3.6 mega bases on the short arm of chromosome 6. The HLA region contains many genes involved in immune function and is characterized as the most polymorphic region in the human genome [1]. As molecular typing technologies have advanced, more than 7,000 HLA alleles have been deposited in the IMGT/HLA Database [2]. The frequency distribution of HLA alleles from diverse human populations has been used as a powerful tool to track human evolutionary processes such as migration, admixture and selection [3]. Genetic variation in the HLA region is associated with many diseases including autoimmune and infectious diseases [1]. Recently, several lines of evidence show that severe and fatal drug hypersensitivity reactions are associated with particular HLA alleles [4]–[6].

In response to the increased needs for large-scale pharmacogenetics association studies, the Japan Pharmacogenomics Data Science Consortium (JPDSC) established a healthy control database including more than 3,000 Japanese volunteers [7]. For successful shared control design, careful matching between cases and controls for their ancestry is needed to avoid inflation of the type I error rate due to population stratification [8]–[10]. In order to control the problem of population stratification, it is required to understand genetic structure underlying the study population.

The origin of modern Japanese has long been debated. It is thought that there are at least two waves of migrations to the Japanese Archipelago. The ancestors of the Jomon people migrated to the Japanese Archipelago in the Upper Paleolithic age (approximately 30,000 years ago). The new migrants, the Yayoi people, came through the Korean Peninsula in the Aeneolithic period (300 BC to 300 AD). The prevailing model for peopling of Japan is the admixture model or “dual structure model” in which modern Japanese was formed by admixture between the Jomon and Yayoi people [11]. Based on morphological studies of teeth and crania, Hanihara proposed that the earlier migrants were from southern Asian lineage whereas the subsequent migrants were from northern Asian lineage [11]. The validity of the admixture hypothesis was partly demonstrated by showing that Japanese populations had close affinity to East Asian populations, especially Korean, and mainland Japanese were located in the middle of Korean and indigenous Japanese populations by the phylogenic analysis [12]. The exhaustive search for the sharing of mitochondrial DNA and Y-Chromosome haplotypes among populations deduced that the ancestors of Jomon people originated from northern and central Asia and the ancestors of Yayoi people came from southern Asia, in contrast to the morphological studies [13]–[16]. The degree of admixture varies across the archipelago, which may influence genetic structure of modern Japanese [16].

Recently, genetic structure of modern Japanese population was examined by using genome-wide single nucleotide polymorphisms (SNPs) data [17]. They found that Japanese individuals were grouped into two clusters: mainland and Okinawa clusters. Furthermore, they found that the HLA region was one of the most differentiated region between mainland and Okinawa clusters [17], [18]. Population genetics studies using HLA alleles have demonstrated that Okinawa have close affinity to Ainu people who are indigenous Japanese and live in northernmost island [19], [20]. Mainland Japanese share a large part of HLA haplotypes with South Korean [21]–[23]. Multiple migration routes to Japan were deduced by examining HLA haplotype distribution among Asian populations at the 2-digit level of resolution [24]. Tokunaga and colleagues pointed out genetic links between East Asians and Native Americans [25].

In this article, we investigated genetic structure of Japanese population by using five-locus HLA genotype data for 2,005 subjects. We examined genetic differentiation among 10 geographical regions across Japan by using the principal component analysis (PCA) and we found a significant level of population substructure in Japanese population. By using a plot of the principal component scores (PCSs), we identified ancestry informative HLA alleles and haplotypes associated with the substructure. We demonstrated that the identified HLA alleles and haplotypes were informative to infer ancestral source populations of Japanese. The results of this study provide evidence to support the admixture model for peopling of Japanese Archipelago.

## Materials and Methods

### Participants

**Figure 1:**
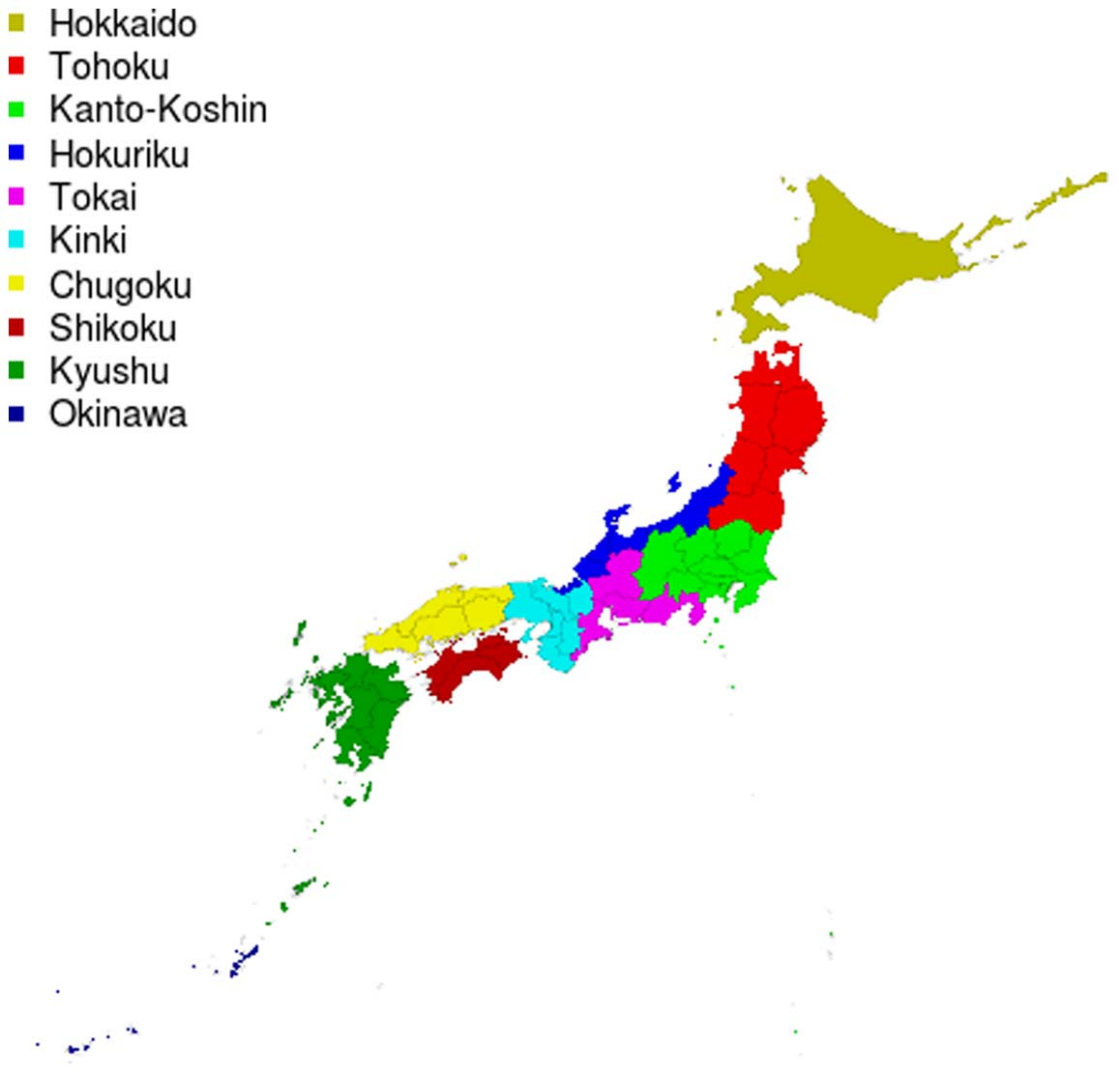
Geographical representation of 10 Japanese regional populations.

The JPDSC collected DNA samples from 2,005 healthy, self-identified Japanese subjects in 10 regions across Japan: Hokkaido, Tohoku, Kanto, Tokai, Hokuriku, Kinki, Chugoku, Shikoku, Kyushu, and Okinawa (Figure 1). Baseline characteristics of the study participants are summarized in Table 1. The ethics committees of GenoDive Pharma Inc. and the JPDSC approved this study. All participants gave written informed consent.

**Table 1:** Baseline characteristics of HLA genotyping for the study participants stratified by district.

District	No. samples	No. observed alleles (Heterozygosity, %)				
		A	B	C	DRB1	DPB1
Hokkaido	120	12 (82.5)	28 (95.8)	14 (93.3)	23 (91.7)	12 (77.5)
Tohoku	198	14 (81.8)	29 (90.4)	15 (86.9)	25 (90.9)	12 (79.8)
Kanto	200	14 (80.0)	31 (92.5)	16 (87.0)	23 (89.5)	11 (77.5)
Hokuriku	115	15 (80.0)	30 (93.0)	16 (90.4)	25 (92.2)	11 (81.2)
Tokai	310	16 (83.9)	35 (92.9)	15 (89.7)	25 (91.6)	13 (78.7)
Kinki	428	17 (78.5)	36 (91.4)	16 (88.5)	28 (92.5)	14 (79.9)
Chugoku	160	14 (80.6)	29 (91.3)	17 (87.5)	26 (91.3)	13 (80.0)
Shikoku	85	12 (83.5)	25 (95.3)	12 (85.9)	23 (91.8)	11 (64.7)
Kyushu	280	18 (83.9)	36 (90.7)	18 (88.2)	27 (87.5)	12 (76.1)
Okinawa	109	11 (79.8)	21 (92.7)	13 (83.5)	21 (89.0)	11 (74.3)

### HLA typing

DNA was extracted from peripheral blood leukocytes by standard methods. We genotyped five HLA loci (*HLA-A*, *-B*, *-C*, *-DRB1*, and *-DPB1*) by using the Luminex assay system and HLA typing kits (WAKFlow HLA typing kits, Wakunaga, Osaka, Japan, or LABType SSO, One Lambda, Canoga Park, CA). In both typing kits, the primers recognizing two polymorphic regions simultaneously were used to reduce allele ambiguities. In the case of allele ambiguity, we adopted the allele combination having the highest frequency in Japanese population. The allele combinations containing an allele with less than 0.005% frequency in Japanese population were excluded in this step. For this filtering, we used the information about HLA allele frequencies in Japanese based on more than 88,000 bone marrow transplantation donors provided by the Central Bone Marrow Data Center in Japan.

### Statistical analysis

The allele frequencies and the heterozygosities for five HLA loci were calculated within each district. *F_st_* statistic was calculated for each pair of regional populations by using the Arlequin version 3.5 [26]. The significance of the genetic distance was evaluated by using 10,000 permutations.

The PCA was performed on the covariance matrix of the normalized allele frequencies [27]. Let ***G*** and ***M*** be the input and normalized data matrices, respectively, with *n* rows and *m* columns, where *n* is the number of populations and *m* is the number of alleles. The element of the normalized matrix is defined as: 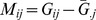, where 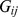 is the frequency of *j*th allele in *i*th population, and 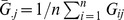. The covariance matrix ***X*** is calculated as:
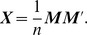



We compute eigenvectors 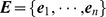 and eigenvalues 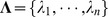 by solving:
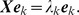



We sought HLA alleles that were associated with the population substructure in terms of PCS. The PCS of *j*th allele for *k*th component is calculated as the linear combinations of the normalized allele frequencies and the eigenvector:
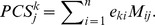



***E*** and ***Λ*** were estimated by using STATA version 11.0 (Stata Inc, College Station, Texas). We hypothesized that the HLA alleles that are associated with the underlying population structure are informative to infer the ancestry of admixed population like Japanese in which degree of admixture is thought to vary across regions. A high value of the absolute PCS is assigned to HLA allele associating with the underlying population structure. For the alleles whose absolute PCS for the first or second component is greater than one standard deviation of PCSs, comparisons of allele frequencies among regional populations were examined by means of Fisher's exact test with R version 2.11.0. We call the identified alleles as “ancestry informative HLA alleles”.

The haplotype phasing was performed via Beagle version 3.3.1 [28]. When examining the haplotype phasing, we separately analyzed Okinawa and the others (referred to as mainland groups) because of possible difference in linkage disequilibrium (LD) structure. The HLA allele and haplotype frequencies in other populations were retrieved from the Allele Frequency Net Database (AFND) [29].

## Results

### Genetic differentiation among 10 regional populations

The numbers of observed HLA alleles and the heterozygosities for five loci were similar across 10 regional populations except for low heterozygosity of *DPB1* locus in Shikoku (Table 1). The heterozygosity of *HLA-DPB1* locus in Shikoku was lower than the others but did not deviate from Hardy-Weinberg equilibrium (*P*>0.05). The degree of genetic diversity within population seems to be similar for each region.

Pair-wise *F_st_* values are shown in Table 2. We found significant differentiations for 17 pairs of regional populations at the nominal significance level (*P*<0.05). Hokuriku was differentiated from five populations (Hokkaido, Tokai, Shikoku, Kyushu, and Okinawa), though the differentiations were not significant after the Bonferroni correction. As expected, Okinawa was highly significantly differentiated from all but Shikoku after the correction for multiple testing (*P*<0.05/45).

**Table 2:** *F_st_* coefficients for pairs of 10 district populations based on the Reynolds' distances by using five HLA loci.

	Tohoku	Kanto	Hokuriku	Tokai	Kinki	Chugoku	Shikoku	Kyushu	Okinawa
Hokkaido	0.00000	0.00008	**0.00214** †	**0.00157** †	0.00045	0.00026	0.00100	0.00037	**0.00983** ¶
Tohoku		0.00000	0.00125	0.00064	0.00000	0.00000	0.00182	0.00086	**0.01070** ¶
Kanto			0.00145	0.00000	0.00000	0.00067	0.00071	0.00017	**0.01127** ¶
Hokuriku				**0.00367** ‡	0.00122	0.00063	**0.00570** ‡	**0.00181** †	**0.01715** ¶
Tokai					0.00057	**0.00170** †	0.00077	**0.00177** ‡	**0.01164** ¶
Kinki						0.00000	0.00069	0.00043	**0.01134** ¶
Chugoku							0.00158	**0.00169** †	**0.01158** ¶
Shikoku								0.00088	**0.00544** ‡
Kyushu									**0.01178** ¶

† Significant difference at the *P*-value<0.05.

‡ Significant difference at the *P*-value<0.01.

¶ Significant difference after the Bonferroni correction at the *P*-value<0.05/45.

### Principal component analysis for the identification of ancestry informative alleles

**Figure 2:**
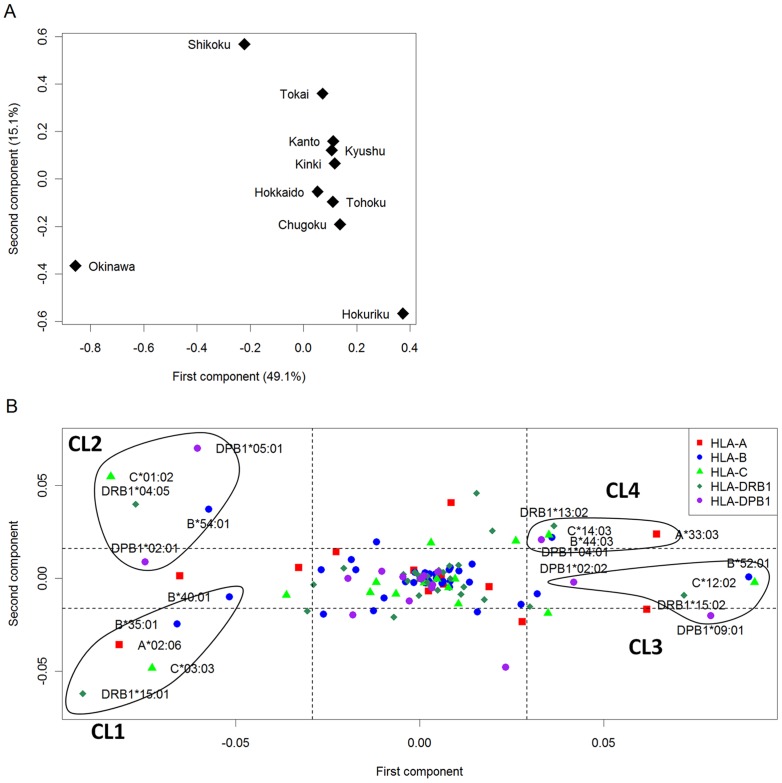
Principal component analysis of 10 regional populations in Japan based on allele frequencies of five HLA loci. A) PCA plot, in which 10 Japanese district populations are plotted according to their corresponding eigenvectors of first and second principal components. B) PCS plot, in which HLA alleles are plotted according to their first and second principal component scores. Dotted lines correspond to mean ± one standard deviation of PCSs. HLA alleles whose absolute PCSs were greater than one standard deviation were selected, followed by Fisher's exact test to evaluate whether the allele frequencies were differentiated among regions. HLA alleles showing significant differentiation at *P*<0.001 are determined as “ancestry informative HLA alleles” and labeled in the plot. The frequency distribution of the identified HLA alleles shows distinct patterns (see Figure 3). The HLA alleles showing similar pattern of differentiation are co-localized in the PCS plot. We marked HLA alleles showing similar patterns of differentiation (referred to as CL1-4) by circles.

Figure 2A shows the result of the PCA of 10 regional populations. Contributions of the first and second components were 49.1% and 15.1%, respectively. Each of the third and subsequent components explained less than 10%. A main cluster including Hokkaido, Tohoku, Kanto, Tokai, Kinki, Chugoku, and Kyushu was formed. The first component was related to the division between Okinawa and mainland groups. The second component seems to explain the variability among mainland groups. Hokuriku and Shikoku were slightly apart from the main cluster. This is consistent with the result shown in Table 2. As a reference, the result of single-locus PCA is shown in Figure S1. In all single-locus PCAs, both ends of the first component were Okinawa and Hokuriku, and Shikoku was closest to Okinawa in terms of the first component, suggesting that the result from the single-locus PCAs reflects the substructure underlying Japanese regional populations.

**Figure 3:**
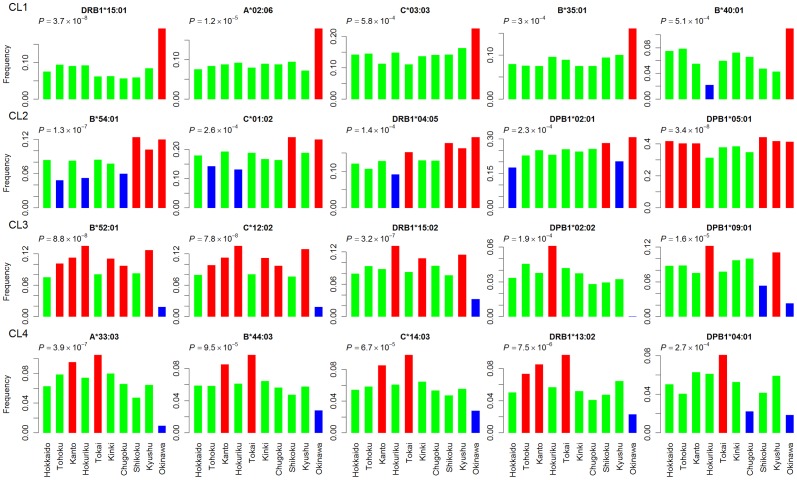
Frequency distribution of HLA alleles associated with population substructure in Japanese. Each row corresponds to a cluster showing similar pattern of allele frequency distribution. Bars are color-coded depending on relative frequencies within each panel: high (red), middle (green), and low (blue). Differences in allele frequency among color-coded two or three classes were examined by means of Fisher's exact test, and the resulting *P*-values are shown.

The HLA alleles are plotted according to the first and second PCSs (Figure 2B). We identified 41 HLA alleles whose absolute PCS for the first or second component was greater than one standard deviation from the mean. Then, we evaluated whether the frequencies of these alleles were remarkably differentiated among regions by means of Fisher's exact test at the significance threshold of *P*<0.001 (<0.05/41). As the result, we identified 20 alleles showing statistically significant differentiation among regions (Figure 3). We classified these alleles into four clusters (referred to as CL1-4) based on the patterns of allele frequency distributions across populations (Figure 3). The first cluster (CL1) including HLA-DRB1*15:01, A*02:06, C*03:03, B*35:01, and B*40:01 was characterized by high frequency in Okinawa (top row in Figure 3). The frequency distributions of HLA-B*54:01, C*01:02, DRB1*04:05, DPB1*02:01, and DPB1*05:01 was characterized by high frequency in Okinawa and Shikoku (CL2, second row in Figure 3). In the CL3 (HLA-B*52:01, C*12:02, DRB1*15:02, DPB1*02:02, and DPB1*09:01; third row in Figure 3), the lowest and highest frequencies were observed in Okinawa and Hokuriku, respectively. The HLA-DPB1*02:02 was frequent in mainland groups (3.8% on average and 6.1% in Hokuriku) but not observed in Okinawa. The frequencies of the alleles in the CL4 were lowest and highest in Okinawa, and Tokai and Kanto, respectively (HLA-A*33:03, B*44:03, C*14:03, DRB1*13:02, and DPB1*04:01; fourth row in Figure 3). This result indicates that a significantly high level of population substructure exists in Japanese based on the HLA alleles, which can lead to false-positive association signals in gene-mapping studies.

It can be seen that the HLA alleles included into the aforementioned clusters are co-localized in the PCS plot (Figure 2B): the CL1, CL2, CL3, and CL4 are located on the bottom-left, left-top, right, and right-top corners, respectively.

### Haplotype reconstruction

The most frequent five-locus HLA haplotypes in mainland groups and Okinawa are shown in Table 3 and Table S1, respectively. The 10 haplotypes explained 19.9% of chromosomes in mainland groups. It can be seen that some of the HLA alleles showing similar pattern in Figure 3 reside on the same haplotypes. The alleles in the CL3 (HLA-C*12:02, B*52:01, DRB1*15:02, and DPB1*09:01) formed the most frequent haplotype (H1). Difference in the H1 haplotype frequency between highest (Hokuriku, 9.13%) and lowest (Okinawa, 1.83%) was statistically significant (*P* = 7.3×10^−4^). All the constituent alleles of the second most common haplotype (H2) were the CL4 alleles. The frequency of the H2 was higher in Tokai (5.16%) and Kanto (4.50%) but not observed in Okinawa (*P* = 8.2×10^−5^). The third most common haplotype (H3) was frequent in Hokuriku (4.78%) and Chugoku (4.69%) and rare in Okinawa (0.46%) (*P* = 1.3×10^−3^). The C*01:02, B*54:01, DRB1*04:05, and DPB1*05:01 in the CL2 formed H4 and H9 haplotypes. At the same time, some of the CL2 alleles appeared on the other haplotypes. For example, C*01:02 associated with B*54:01 and DRB1*04:05 on the H4 and H9, but also associated with B*46:01 and DRB1*08:03 on the H5 and H7. The alleles in the CL1 did not form common haplotypes.

**Table 3:** The 10 most common five-locus HLA haplotypes in mainland Japanese.

Haplotype						Frequency (%)										
ID	A	C	B	DRB1	DPB1	Mainland†	Hokkaido	Tohoku	Kanto	Hokuriku	Tokai	Kinki	Chugoku	Shikoku	Kyushu	Okinawa
H1	24:02	12:02	52:01	15:02	09:01	6.38	5.42	5.81	4.50	9.13	5.16	7.01	6.56	3.53	8.57	1.83
H2	33:03	14:03	44:03	13:02	04:01	3.38	2.08	3.28	4.50	2.61	5.16	3.15	1.56	1.76	3.39	0.00
H3	24:02	07:02	07:02	01:01	04:02	2.82	3.75	2.78	3.50	4.78	1.61	2.57	4.69	2.94	1.79	0.46
H4	24:02	01:02	54:01	04:05	05:01	2.40	2.92	1.26	2.25	0.87	3.55	2.45	1.56	1.76	3.04	3.67
H5	02:07	01:02	46:01	08:03	05:01	0.95	0.42	1.01	0.25	0.43	0.48	1.29	0.94	1.76	1.61	0.92
H6	33:03	14:03	44:03	13:02	02:01	0.90	1.25	1.01	1.00	0.43	1.94	0.58	0.63	0.59	0.36	0.00
H7	02:07	01:02	46:01	08:03	02:02	0.79	0.42	1.01	0.25	0.87	1.13	1.05	0.31	0.00	0.89	0.00
H8	11:01	04:01	15:01	04:06	02:01	0.79	0.00	1.52	0.25	0.00	0.97	1.52	0.94	0.00	0.18	0.00
H9	11:01	01:02	54:01	04:05	05:01	0.74	0.83	0.25	0.50	0.43	0.16	0.70	0.63	3.53	1.25	0.00
H10	24:02	12:02	52:01	15:02	05:01	0.71	0.00	0.51	1.50	0.87	0.48	0.58	0.31	1.76	0.89	0.00

† Nine mainland groups (Hokkaido, Tohoku, Kanto, Hokuriku, Tokai, Kinki, Chugoku, Shikoku, and Kyushu) were combined.

It is well known that the recombination hot spots exist within the MHC especially between *HLA-DRB1* and *HLA-DPB1* [30], [31]. We reconstructed four-locus haplotypes excluding *HLA-DPB1* locus in order to examine whether the haplotype reconstruction for regions crossing the recombination hot spots affected our results. The most common four-locus HLA haplotypes in mainland Japan and Okinawa are represented in Tables S2 and S3, respectively. The most frequent four-locus haplotypes correspond approximately to the most frequent five-locus haplotypes, indicating that LD maintained in the most frequent haplotypes to some extent and our results were not affected by the recombination hot spots.

### Linkage disequilibrium analysis and searching for shared ancestry

**Figure 4:**
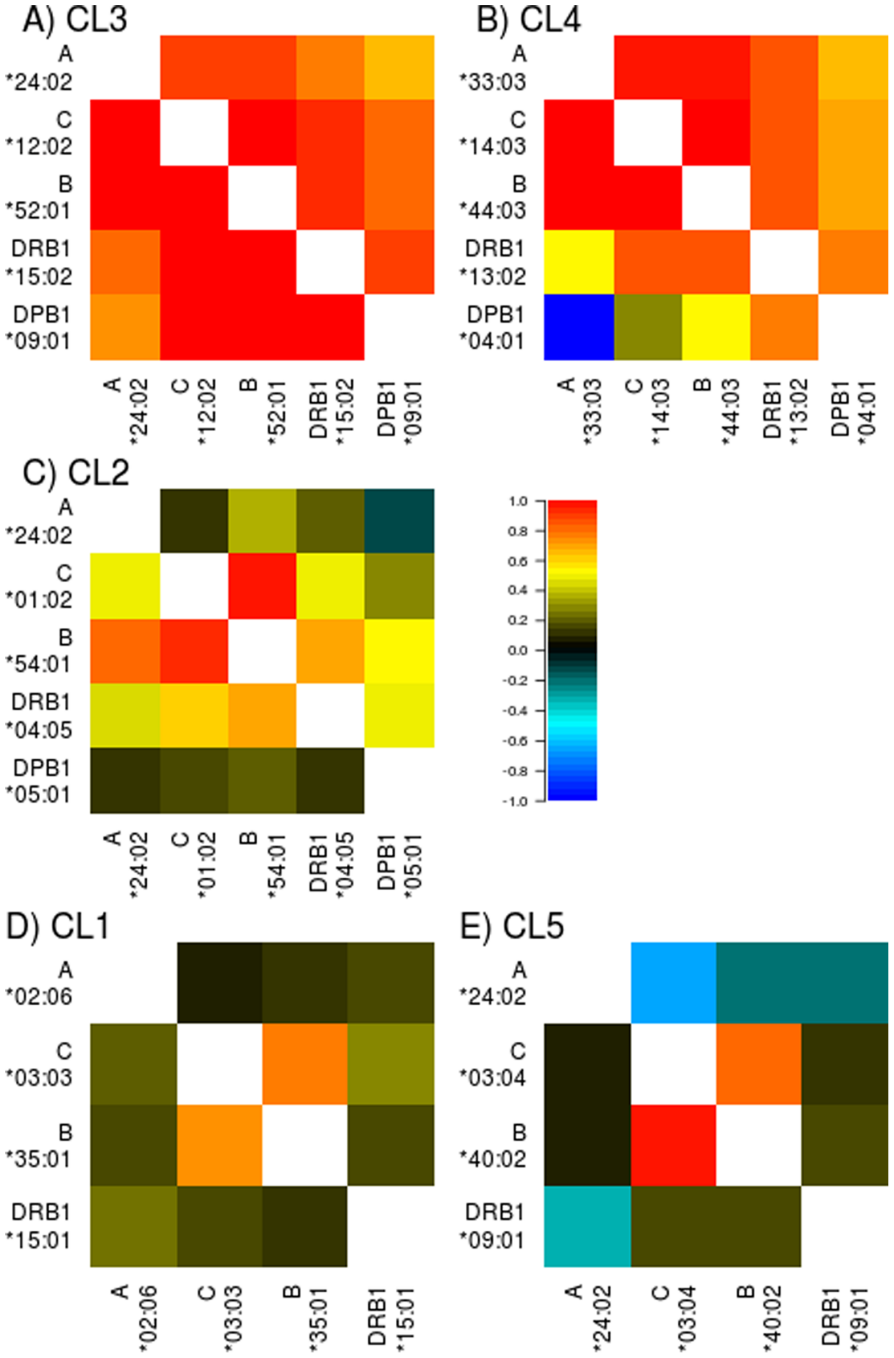
Extent of linkage disequilibrium (*D*′) between pairs of HLA alleles in the same cluster represented in **Figure 3**. The values above and below diagonal elements correspond to *D*′ values estimated in mainland and Okinawa groups, respectively.

We examined the extent of LD between pairs of HLA alleles on the identified haplotypes in terms of pair-wise *D*′ [32]. The extent of LD between pairs of HLA alleles in each cluster is shown in Figure 4. For each pair, the extent of LD in mainland was similar to that in Okinawa. The *D*′ values were high for pairs of HLA alleles in the CL3 and CL4 (Figure 4A and 4B). The intermediate level of the *D*′ values were observed for pairs of alleles in the CL2 (Figure 4C). The LD was weak for pairs of alleles in the CL1 (Figure 4D). Interestingly, the extent of LD was stronger for pairs of HLA alleles characterized by low frequency in Okinawa than those characterized by high frequency in Okinawa.

We hypothesized that the LD across HLA alleles characterized by low frequency in Okinawa (CL3 and CL4) are strong because HLA haplotypes carrying these alleles had been recently derived from the Korean Peninsula and expanded in Japan's mainland rapidly. Thus, we examined genetic relationship between Japanese and South Korean. We compared the frequencies of haplotypes carrying A*33:03 allele that was characterized by high frequency in South Korean (Table 4). There were four common haplotypes carrying A*33:03 in South Korean. While the haplotype A*33:03-B*44:03-C*14:03-DRB1*13:02 was frequent in mainland Japanese and South Korean, the other three haplotypes were frequent in South Korean but rare or absent in Japanese. By searching the AFND database, the haplotype A*33:03-B*44:03-C*14:03-DRB1*13:02 was observed only in Japanese and Korean. In contrast, the other three haplotypes were prevalent in East and Southeast Asian populations (Table 4) [21], [33]–[36]. The haplotype A*24:02-C*12:02-B*52:01-DRB1*15:02 was observed only in Japanese and South Korean. These findings may reinforce our hypothesis that the origin of H1 and H2 haplotypes was the Korean Peninsula.

**Table 4:** Comparison of haplotype frequencies containing A*33:03 allele observed in South Korean to mainland Japanese, Okinawa Japanese and other East and Southeast Asian populations.

Haplotype	Frequency (%)†						
	SouthKorean	MainlandJapanese	OkinawaJapanese	ChinaDai	YunnanHan Chinese	Taiwanese	Vietnamese
A*33:03-B*44:03-C*07:01/07:06-DRB1*07:01	2.99	0.05	0.00	-	-	0.71	2.60
A*33:03-B*44:03-C*14:03-DRB1*13:02	4.23	5.01	0.46	-	-	-	-
A*33:03-B*58:01-C*03:02-DRB1*03:01	1.85	0.08	0.00	4.40	3.00	3.91	3.50
A*33:03-B*58:01-C*03:02-DRB1*13:02	2.99	0.40	0.00	2.40	3.00	2.14	1.20

† “-”, not reported in literatures because the haplotype was rare or absent.

The haplotypes H4, H5, H7 and H9 bear C*01:02. The strength of LD between pairs of constituent alleles of these haplotypes was not so strong compared to the H1 and H2. The fragment of the H4 and H9 haplotypes (B*54:01-DRB1*04:05) was found in South Korean (2.5%), the Ivatan people in Philippines (1.0%), and the Siraya people in Taiwan (2.9%). The common segment of the H5 and H7 haplotypes (A*02:07-C*01:02-B*46:01-DRB1*08:03) was found in the Nu and Jinuo people in the Yunnan province of China (4.3% and 2.6%) [37], [38]. The fragment of the H5 and H7 haplotypes (B*46:01-DRB1*08:03) was observed in South Korean (2.6%), the Minnan people in Taiwan (2.5%), and the Pazeh people in Taiwan (1.8%). The sharing of these haplotypes indicates that modern Japanese is also affected by southern part of East Asian lineage.

We sought shared ancestry of the alleles in the CL1. The alleles with higher frequency in Okinawa (CL1) did not form common haplotypes. In Ainu people who were descendants of indigenous Japanese, some of the alleles were frequent (A*02:06, 20.0%; B*35:01, 11.0%) but the others were not so frequent (B*40:01, 6.0%; and DRB1*15:01, 2.0%) [20]. We scrutinized the prevalence of haplotypes carrying alleles that were frequent in Okinawa and Ainu and found that the haplotype A*02:06-B*35:01 was frequent in the Yupik people in Alaska (2.9%) [39].

**Figure 5:**
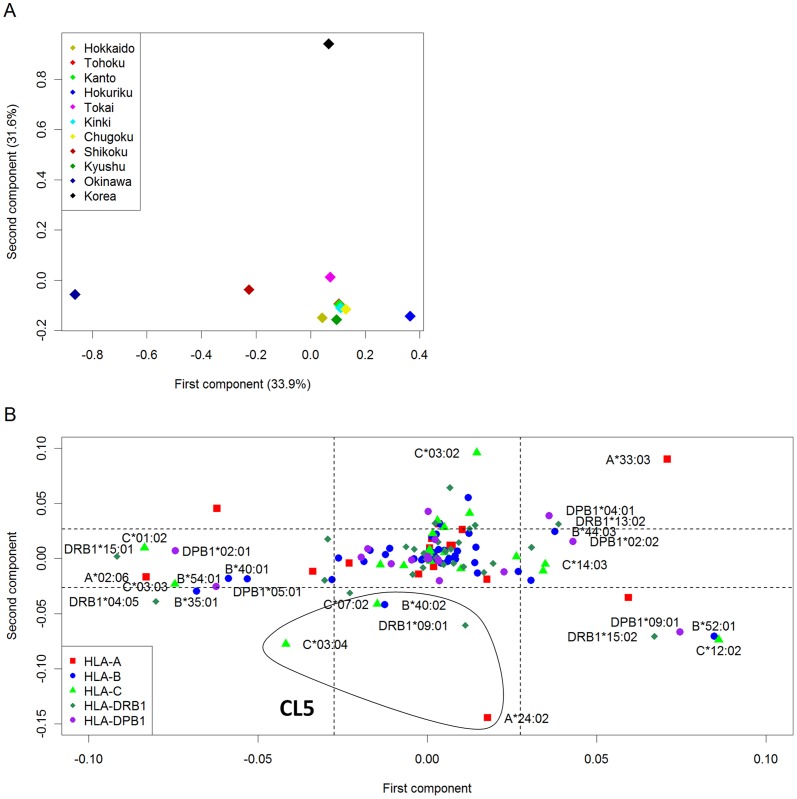
Principal component analysis of Japanese and South Korean. A) PCA plot. B) PCS plot. The allele frequencies of South Korean were retrieved from the literatures [21], [61]. Dotted lines correspond to mean ± one standard deviation of PCSs. HLA alleles that are labeled and in a circle shows high frequency in Japanese but low frequency in South Korean (referred to as cluster 5 [CL5]; A*24:02, C*03:04, C*07:02, B*40:02, and DRB1*09:01). Alleles shown in Figure 3 are also labeled.

Finally, we performed the PCA approach including both Japanese and South Korean to identify the alleles that were differentiated between these populations (Figure 5). The first and second components explained 33.9% and 31.6% of variability, respectively. The first component distinguished between mainland and Okinawa. The second component captured differentiation between Japanese and South Korean (Figure 5A). According to the PCS plot (Figure 5B), we can find highly differentiated alleles between Japanese and South Korean at either end of the second component (e.g., HLA-C*03:02, A*24:02, and A*33:03). We focused on the alleles located in the middle of the bottom half of Figure 5B (A*24:02, C*03:04, C*07:02, B*40:02, and DRB1*09:01; referred to as CL5), which were characterized by higher frequency in Japanese compared to South Korean. Among them, C*03:04 and B*40:02 were in LD (*D′* = 0.940 and 0.755 in Okinawa and mainland, respectively) (Figure 4E). The haplotype C*03:04-B*40:02 were frequent in mainland Japanese (6.30%) and Okinawa (8.72%) but infrequent in South Korean [21]. Therefore, we searched the prevalence of this haplotype in the AFND database (Table 5). The C*03:04-B*40:02 haplotype was observed in Aleuts (Bering Island [40]), Eskimos (Alaskan Yupik [39]), North-American Amerindians, Meso-American Amerindians (Tarahuara, Mixe, Mixtec, and Zapotec in Mexico [41], [42]), Taiwanese (Minnan), Taiwan's aborigines (Tao, Ami, Paiwan, and Siraya), and Philippine aborigines (Ivatan). For Taiwan's populations except for the Tao people, the C*03:04-B*40:02 haplotype frequencies were not so high although the frequencies of C*03:04 and B*40:02 were high, indicating the difference in the LD structure (Table 5). The Tao (or Yami) people live on the Orchid Island off the east coast of Taiwan, and therefore are considered to be genetically isolated from the other Taiwan's aborigines [43]. It is well known that the Tao and Ivatan people have close affinities in terms of genetic and linguistic characteristics [44]. The Tao was the only population among Taiwan's aborigines who had the haplotype *A24-Cw10-B61* that was the serological equivalent encoded by A*24:02-C*03:04-B*40:02 and commonly observed in the Orochon, Mongolians, Inuit, Yakut, and Buryats [43]. The frequencies of A*24:02-C*03:04-B*40:02 haplotype were 2.41% and 3.21% in mainland and Okinawa, respectively. The aforementioned Aleuts, Eskimos and Amerindian populations carried A*24:02-C*03:04-B*40:02 at the high frequencies ranging from 1.9% to 6.9% (Table 5). These results suggest shared ancestry of early Japanese with the ancestral northern Asian lineage who crossed the Bering Strait land bridge and became founder population of the Native Americans.

**Table 5:** Prevalence of C*03:04-B*40:02 haplotypes.

Population	C*03:04	B*40:02	C*03:04- B*40:02
Mainland, Japan†	12.63	8.02	6.30
Okinawa Island, Japan†	16.51	9.17	8.72
South Korea	3.90	3.80	-¶
Yupik in Alaska, USA†	38.60	29.60	27.20
Aleut in Bering Island, Russia†	20.00	15.30	15.30
North American native, USA†	11.20	5.90	3.70
Tarahumara in Chihuahua, Mexico†	39.80	20.50	13.07
Mixe in Oaxaca, Mexico	21.70	11.30	3.90
Mixetec in Oaxaca, Mexico	4.00	6.90	3.00
Zapotec in Oaxaca, Mexico	10.40	3.70	2.20
Hispanic, USA	7.30	5.10	3.80
Tao, Taiwan‡	21.00	23.00	15.00
Ami, Taiwan	12.20	5.10	1.50
Paiwan, Taiwan	52.00	13.70	3.20
Siraya, Taiwan	20.60	19.60	2.90
Minnan, Taiwan	14.20	3.90	2.50
Ivatan, Philippine	9.00	14.00	1.00

† A*24:02-C*03:04- B*40:02 haplotype was found in these populations.

‡ *A24-Cw10-B61* haplotype was found in these populations.

¶ “-”, not reported in literatures because the haplotype was rare or absent.

## Discussion

Population stratification is a potential cause of the inflation of false positive findings in genetic association studies. We demonstrated that there was a substantial level of population stratification in Japanese population, especially between Okinawa and other mainland groups. Therefore, careful consideration on population substructure is needed in genetic association studies in Japanese population. It is recommended that case-control study is performed by stratifying into two groups (mainland and Okinawa), followed by meta-analysis integrating the results from the two groups [17], [45]–[47]. To a lesser extent, there were differences in frequencies of HLA alleles and haplotypes among mainland groups. In order to examine extent of population substructure among mainland groups, we performed another PCA after removing Okinawa from the dataset (Figure S2). In the first component, both ends of the first component were Shikoku and Hokuriku. In the first component of the PCA including all the Japanese populations (Figure 2A), both ends were Okinawa and Hokuriku, and Shikoku was closest to Okinawa. This result shows the localization of mainland populations in the PCA plot (Figure S2) is similar to that in Figure 2A regarding the first component, implying that the population stratification exists among mainland populations. A large scale study is needed to corroborate the differentiations among mainland groups.

We identified HLA alleles which contribute to the underlying population substructure by using a PCA-based method. We performed a “two-step” procedure to detect ancestry informative HLA alleles. First, we selected HLA alleles whose absolute PCSs for the first or second component were greater than one standard deviation from the mean. Second, we identified HLA alleles showing significant differentiation across regions. The main advantage of the two-step procedure against a simple one-step procedure without the PCS-based step is that a large proportion of undifferentiated HLA alleles can be filtered out, and therefore we can remarkably reduce the number of statistical tests examined. Indeed, about 70% of the HLA alleles were filtered out in the first step (out of 140, only 41 alleles were statistically tested). Additionally, the PCS plot itself is a powerful tool for population genetics studies. In the PCS plot, the alleles with similar pattern of frequency differentiation among populations are co-localized as shown in Figure 2B and 5B. Thus, it is useful to characterize a set of alleles associated with differentiation among the populations analyzed.

The novel finding of this study is that the alleles characterized by high frequency in mainland Japanese compared to Okinawa formed five-locus haplotypes and the constituent alleles showed strong LDs; on the other hand, the alleles with higher frequency in Okinawa compared to mainland showed decayed LDs. The haplotypes H1 and H2, whose constituent alleles were in strong LD, were found only in Japanese and South Korean. It is plausible that if a haplotype is derived and goes through rapid expansion, its constituent alleles will show strong LD [48]–[50]. Therefore, it is suggested that these haplotypes had been generated in the Korean Peninsula and was carried over into Japan's mainland followed by the rapid expansion probably at the Yayoi period. The haplotypes whose constituent alleles were in the intermediate levels of LD were shared by south East Asian populations.

The ten most frequent five-locus HLA haplotype made up only 19.9% of chromosomes in mainland Japanese, implying that the decay of LD generated segmented haplotypes during a long period of isolation of the Japanese population. The alleles characterized by high frequency in Okinawa (CL1) and by high frequency in Japanese compared to South Korean (CL5) showed lower levels of LD as depicted in Figure 4D and 4E, respectively, and did not form common five-locus haplotypes. Therefore, consideration on segmented haplotypes seems to be a straightforward approach to infer shared ancestry of prehistoric Japanese. The haplotype A*24:02-C*03:04-B*40:02 was observed in Japanese, Aleuts, Eskimos, North-American Amerindians and Meso-American Amerindians. The *A24-Cw10-B61* haplotype, the serological equivalent encoded by A*24:02-C*03:04-B*40:02, was also frequent in Orchid Island in Taiwan, Mongol, Siberia and Arctic regions [43]. These findings suggest that the haplotype A*24:02-C*03:04-B*40:02 had been derived from early Japanese (Jomon people) who existed prior to the migration wave from the Korean Peninsula and this haplotype is one of the genetic footprints of the migration route of prehistoric ancient population from Asia to the New World.

The origin of East Asian has long been debated. The study based on genome-wide SNPs support the hypothesis that a single wave of migration coming from southern route populated East Asian populations [51]. Another hypothesis known as “pincer model” of a separate migratory route from Central Asia together with southern route has been proposed for the origin of East Asian populations [52], [53]. Recent studies based on HLA alleles demonstrate that the pincer model fit better [54]. The population entered Siberia by 45-40 thousand years ago (ka), and the offshoots of the population gave rise to early Japanese population [55]. The whole-genome sequencing of permafrost-preserved hair from an ancient individual in Greenland demonstrated that early modern human who entered the New World was Asian rather than European [56]. It is thought that the first people crossed the Bering Strait land bridge to America by 15 ka. Recent genome-wide SNP study shows that the “First American” ancestry distributed through Native Americans but two additional waves of gene flow affected Eskimo-Aleut populations in the Arctic region and Na-Dene-speaking population in Canada [57]. Some authors identified genetic variants shared between Eurasia and North America [58]. These findings fit our result, suggesting that the haplotype A*24:02-C*03:04-B*40:02 originated from Asia and diverged through the North to Central America by the “First American”.

The fact that Japanese have the haplotype, which was not detected in the Chinese and Korean but dispersed through the migration route of Americans, suggests prehistoric shared ancestry of Japanese with Northern Asian lineage. It is possible that East Asian populations including Chinese and Korean had shared this haplotype at the prehistoric age. During a long period, the haplotype might have disappeared from East Asians except for the isolated populations, Japanese and the Tao people in Orchid Island of Taiwan. At the same time, we detected the haplotypes whose constituent alleles are tightly linked, indicating the recent gene flow from the Korean Peninsula. There are two possible migration routes of the haplotypes whose constituent alleles show intermediate levels of LD: i) northern route through the Korean Peninsula or ii) southern route through Taiwan. If the latter is true, modern Japanese descend from at least three waves of migration from Asia. These results may support the admixed model for the peopling of Japan.

Current population genetics studies using genotyping of HLA alleles at the four-digit level of resolution rely on the technology that is focused only on the most polymorphic regions (exons 2 and 3 for class I genes and exon 2 for class II genes). Next generation sequencing technologies enable us to more high resolution typing of HLA alleles [59]. The high resolution HLA sequencing will accelerate studies for tracing human evolutionary process by investigating genealogical relationships among HLA haplotypes [60].

## Supporting Information

Figure S1 Principal component analysis of 10 regional populations in Japan based on allele frequencies for each HLA locus.
(TIFF)

Figure S2 Principal component analysis of 9 mainland populations based on allele frequencies of five HLA loci.
(TIFF)

Table S1 The 10 most common five-locus HLA haplotypes in Okinawa.
(DOCX)

Table S2 The 10 most common four-locus HLA haplotypes in mainland Japanese.
(DOCX)

Table S3 The 10 most common four-locus HLA haplotypes in Okinawa.
(DOCX)
