# Supplementary figures and images for: Detection of Ancestry Informative HLA Alleles Confirms the Admixed Origins of Japanese Population
Source: PLoS One. 2013 Apr 5;8(4):e60793. doi: 10.1371/journal.pone.0060793 (PMC3618337; doi:10.1371/journal.pone.0060793)

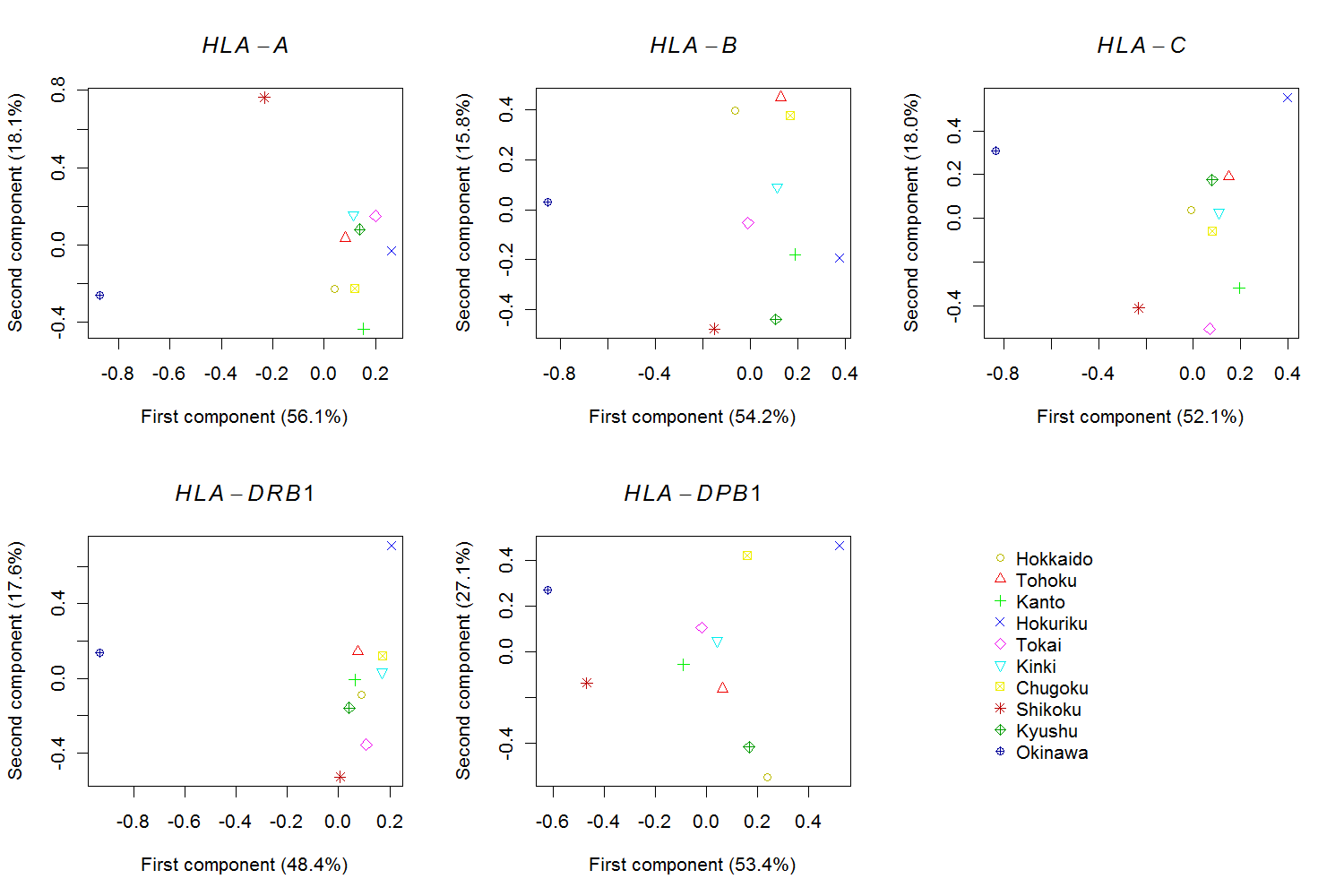

Supplement: Figure S1 — Principal component analysis of 10 regional populations in Japan based on allele frequencies for each HLA locus. (TIFF) [file pone.0060793.s001.tiff]

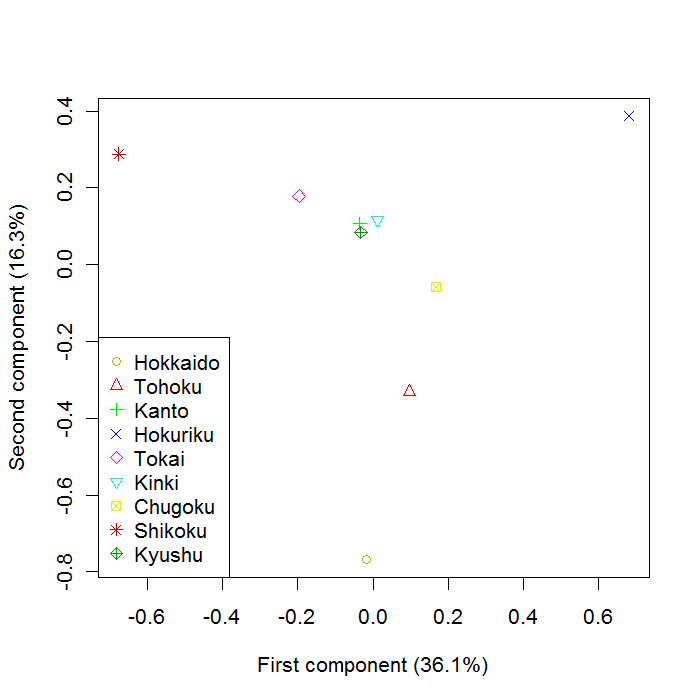

Supplement: Figure S2 — Principal component analysis of 9 mainland populations based on allele frequencies of five HLA loci. (TIFF) [file pone.0060793.s002.tiff]
