# Supplementary material for: Detection of Ancestry Informative HLA Alleles Confirms the Admixed Origins of Japanese Population
Source: PLoS One. 2013 Apr 5;8(4):e60793. doi: 10.1371/journal.pone.0060793 (PMC3618337; doi:10.1371/journal.pone.0060793)
Supplement: Table S1 — The 10 most common five-locus HLA haplotypes in Okinawa. (DOCX) [file pone.0060793.s003.docx]

Table S1. The 10 most common five-locus HLA haplotypes in Okinawa.

| Haplotype | | | | | Frequency (%) |
| --- | --- | --- | --- | --- | --- |
| A | C | B | DRB1 | DPB1 |  |
| 24:02 | 01:02 | 54:01 | 04:05 | 05:01 | 3.67 |
| 24:02 | 01:02 | 54:01 | 04:05 | 19:01 | 1.83 |
| 24:02 | 12:02 | 52:01 | 15:02 | 09:01 | 1.83 |
| 24:02 | 01:02 | 59:01 | 04:05 | 05:01 | 1.83 |
| 24:02 | 01:02 | 54:01 | 04:05 | 02:01 | 1.38 |
| 02:06 | 08:03 | 48:01 | 15:01 | 02:01 | 1.38 |
| 02:01 | 03:04 | 40:02 | 08:02 | 04:02 | 1.38 |
| 02:06 | 03:03 | 35:01 | 15:01 | 02:01 | 1.38 |
| 24:02 | 03:03 | 35:01 | 14:05 | 05:01 | 1.38 |
| 24:02 | 03:03 | 35:01 | 15:01 | 02:01 | 0.92 |
