# Supplementary material for: Detection of Ancestry Informative HLA Alleles Confirms the Admixed Origins of Japanese Population
Source: PLoS One. 2013 Apr 5;8(4):e60793. doi: 10.1371/journal.pone.0060793 (PMC3618337; doi:10.1371/journal.pone.0060793)
Supplement: Table S2 — The 10 most common four-locus HLA haplotypes in mainland Japanese. (DOCX) [file pone.0060793.s004.docx]

Table S2. The 10 most common four-locus HLA haplotypes in mainland Japanese.

| Haplotype | | | |  | Frequency (%) | | | | | | | | | | |
| --- | --- | --- | --- | --- | --- | --- | --- | --- | --- | --- | --- | --- | --- | --- | --- |
| A | C | B | DRB1 |  | Mainland^†^ | Hokkaido | Tohoku | Kanto | Hokuriku | Tokai | Kinki | Chugoku | Shikoku | Kyushu | Okinawa |
| 24:02 | 12:02 | 52:01 | 15:02 |  | 8.10 | 5.83 | 7.58 | 7.00 | 10.87 | 6.77 | 8.76 | 7.81 | 5.88 | 10.36 | 1.83 |
| 33:03 | 14:03 | 44:03 | 13:02 |  | 5.01 | 3.33 | 5.56 | 6.75 | 3.48 | 7.58 | 4.44 | 3.13 | 3.53 | 4.29 | 0.92 |
| 24:02 | 07:02 | 07:02 | 01:01 |  | 3.98 | 4.58 | 3.28 | 4.25 | 5.22 | 2.42 | 4.67 | 6.88 | 2.94 | 2.86 | 0.46 |
| 24:02 | 01:02 | 54:01 | 04:05 |  | 3.11 | 2.92 | 1.52 | 3.00 | 0.87 | 4.35 | 3.27 | 2.81 | 2.94 | 3.93 | 6.88 |
| 02:07 | 01:02 | 46:01 | 08:03 |  | 2.19 | 1.25 | 2.78 | 1.25 | 1.74 | 1.77 | 2.69 | 1.88 | 2.94 | 2.68 | 0.92 |
| 11:01 | 04:01 | 15:01 | 04:06 |  | 1.27 | 0.83 | 2.02 | 0.25 | 0.00 | 2.10 | 1.99 | 1.88 | 0.00 | 0.18 | 0.46 |
| 11:01 | 01:02 | 54:01 | 04:05 |  | 0.98 | 0.83 | 0.25 | 0.75 | 0.87 | 0.65 | 0.70 | 0.63 | 2.94 | 2.14 | 0.46 |
| 24:02 | 08:01 | 40:06 | 09:01 |  | 0.84 | 1.25 | 1.52 | 1.00 | 2.17 | 1.29 | 0.23 | 0.00 | 0.59 | 0.54 | 0.00 |
| 31:01 | 14:02 | 51:01 | 08:02 |  | 0.82 | 0.83 | 1.26 | 0.50 | 1.30 | 0.81 | 0.58 | 1.25 | 0.59 | 0.71 | 0.46 |
| 24:02 | 01:02 | 59:01 | 04:05 |  | 0.79 | 1.67 | 1.52 | 0.50 | 0.87 | 0.81 | 0.70 | 0.63 | 1.18 | 0.18 | 3.67 |

^†^Nine mainland groups (Hokkaido, Tohoku, Kanto, Hokuriku, Tokai, Kinki, Chugoku, Shikoku, and Kyushu) were combined.
