# Supplementary material for: Detection of Ancestry Informative HLA Alleles Confirms the Admixed Origins of Japanese Population
Source: PLoS One. 2013 Apr 5;8(4):e60793. doi: 10.1371/journal.pone.0060793 (PMC3618337; doi:10.1371/journal.pone.0060793)
Supplement: Table S3 — The 10 most common four-locus HLA haplotypes in Okinawa. (DOCX) [file pone.0060793.s005.docx]

Table S3. The 10 most common four-locus HLA haplotypes in Okinawa.

| Haplotype | | | | Frequency (%) |
| --- | --- | --- | --- | --- |
| A | C | B | DRB1 |  |
| 24:02 | 01:02 | 54:01 | 04:05 | 6.88 |
| 24:02 | 01:02 | 59:01 | 04:05 | 3.67 |
| 02:01 | 15:02 | 51:01 | 15:01 | 1.83 |
| 24:02 | 12:02 | 52:01 | 15:02 | 1.83 |
| 02:01 | 03:04 | 40:02 | 08:02 | 1.38 |
| 02:07 | 14:03 | 44:03 | 13:02 | 1.38 |
| 11:01 | 07:02 | 39:01 | 15:01 | 1.38 |
| 02:01 | 03:04 | 40:01 | 14:54 | 0.92 |
| 02:01 | 07:02 | 07:02 | 01:01 | 0.92 |
| 02:01 | 07:02 | 39:01 | 08:03 | 0.92 |
